# Biochemical Characterization of Enzyme Fidelity of Influenza A Virus RNA Polymerase Complex

**DOI:** 10.1371/journal.pone.0010372

**Published:** 2010-04-29

**Authors:** Shilpa Aggarwal, Birgit Bradel-Tretheway, Toru Takimoto, Stephen Dewhurst, Baek Kim

**Affiliations:** Department of Microbiology and Immunology, University of Rochester Medical Center, Rochester, New York, United States of America; University of Hong Kong, Hong Kong

## Abstract

**Background:**

It is widely accepted that the highly error prone replication process of influenza A virus (IAV), together with viral genome assortment, facilitates the efficient evolutionary capacity of IAV. Therefore, it has been logically assumed that the enzyme responsible for viral RNA replication process, influenza virus type A RNA polymerase (IAV Pol), is a highly error-prone polymerase which provides the genomic mutations necessary for viral evolution and host adaptation. Importantly, however, the actual enzyme fidelity of IAV RNA polymerase has never been characterized.

**Principal Findings:**

Here we established new biochemical assay conditions that enabled us to assess both polymerase activity with physiological NTP pools and enzyme fidelity of IAV Pol. We report that IAV Pol displays highly active RNA-dependent RNA polymerase activity at unbiased physiological NTP substrate concentrations. With this robust enzyme activity, for the first time, we were able to compare the enzyme fidelity of IAV Pol complex with that of bacterial phage T7 RNA polymerase and the reverse transcriptases (RT) of human immunodeficiency virus (HIV-1) and murine leukemia virus (MuLV), which are known to be low and high fidelity enzymes, respectively. We observed that IAV Pol displayed significantly higher fidelity than HIV-1 RT and T7 RNA polymerase and equivalent or higher fidelity than MuLV RT. In addition, the IAV Pol complex showed increased fidelity at lower temperatures. Moreover, upon replacement of Mg^++^ with Mn^++^, IAV Pol displayed increased polymerase activity, but with significantly reduced processivity, and misincorporation was slightly elevated in the presence of Mn^++^. Finally, when the IAV nucleoprotein (NP) was included in the reactions, the IAV Pol complex exhibited enhanced polymerase activity with increased fidelity.

**Significance:**

Our study indicates that IAV Pol is a high fidelity enzyme. We envision that the high fidelity nature of IAV Pol may be important to counter-balance the multiple rounds of IAV genome amplification per infection cycle, which provides IAV Pol with ample opportunities to generate and amplify genomic founder mutations, and thus achieve optimal viral mutagenesis for its evolution.

## Introduction

The high genetic diversity of influenza A virus (IAV), together with gene reassortment, forms viral quasispecies which enable IAV to acquire its efficient host adaptability. It is widely accepted that the efficient evolutionary capacity of IAV is facilitated by its highly error-prone replication process [1], [2]. Every genome copy packaged in newly produced viral progenies is synthesized exclusively by the IAV RNA polymerase (Pol), and thus it has been logically assumed that IAV Pol is a highly error-prone polymerase which provides the genomic mutations necessary for viral evolution and host adaptation. Importantly, however, the actual enzyme fidelity of IAV RNA polymerase, which is the main driving force for viral mutation synthesis, has never been characterized. On the other hand, human immunodeficiency virus type 1 (HIV-1) is also well known for its genomic hypermutability [3], [4], and indeed HIV-1 DNA polymerase, called reverse transcriptase (RT), is the most error prone DNA polymerase among those involved in genome replication [5]. However, the replication strategy of these polymerases is very different. HIV-1 RT replicates viral genomes only twice per infection cycle, and every RNA genome in the HIV-1progeny virions is actually produced by host RNA polymerase II. In contrast, IAV Pol not only is responsible for synthesizing every viral RNA genome but also amplifies viral RNAs multiple times per infection cycle, which gives IAV Pol ample chances to generate mutations in every infection event.

IAV Pol has been qualitatively studied for its RNA-dependent RNA polymerase activity using various biochemical settings [6], [7], [8]. However, the mechanistic studies of IAV Pol complex, which determine basic kinetic parameters of the polymerases such as the nucleotide binding affinity, conformational change and chemical catalysis, have not been explored. This is because of several technical difficulties that have hindered this type of biochemical analysis for IAV Pol. First, the expression and purification of IAV Pol has been less than optimal for producing a large quantity of the active enzyme, simply because it is technically more challenging to express and purify the IAV Pol complex which consists of three different subunits, PA, PB1 and PB2, as compared to other single subunit polymerases such as HIV-1 RT. Second, polymerase studies which determine the basic kinetic parameters of DNA/RNA polymerases employ pre-steady state conditions instead of the steady state condition [9], [10]. However, since the pre-steady state condition requires the saturation of the template/primer complex used (100–200 nM) by the polymerase, a large amount of the purified polymerase is necessary for this type of analysis. Third, the IAV Pol assays use very high concentrations (mM) of primers such as ApG and cellular mRNAs, while other polymerase assays (i.e. HIV-1 RT) employ much lower concentrations of the primers (10–100 nM). Therefore, under the current IAV Pol reaction conditions available, only a fraction of the total primer is extended in each polymerase reaction, and thus the use of the 5′ end labeled primers, which are routinely used in kinetic assays of other polymerases, are not easily applicable to the IAV Pol assay.

Most frequently used polymerization assays of IAV Pol in current studies are gel-based standing start primer extension reactions that incorporate NTP substrates using ApG or cellular mRNAs as primers. In typical IAV Pol assays, one of the four NTPs is an α-^32^P-labeled radioactive NTP (i.e. α-^32^P-GTP), which aids in the visualization of the extended products, and the other three NTPs are nonradioactive (i.e. ATP, CTP and UTP), [6], [7], [8], [11], [12]. However, while the three nonradioactive NTPs are normally used at mM ranges, the concentration of the radioactive NTP (i.e. α-^32^P-GTP) is relatively very low (0.08 to 50 µM), simply because the highly concentrated radioactive NTP is not readily available, and use of a large quantity of radioactivity is technically challenging. Indeed, many recent reports that studied the steady state kinetic NTP incorporation by IAV Pol still used approximately 1/100 to 1/1000 biased nucleotide pools for the radioactive NTP (i.e. GTP) to the other three nonradioactive NTPs (i.e. ATP, CTP and UTP) [8], [13], [14]. These unbalanced NTP pools in the reaction raise several concerns. First, it is highly likely that the use of 100- or 1000-fold lower concentration of the radioactive GTP, as compared to the three other nonradioactive NTPs, induces a serious polymerase kinetic bias: the entire IAV Pol reaction rate is simply dependent on the limited availability of radioactive GTP in the reaction, ultimately leading to the underestimation of the kinetic activity of IAV Pol. Second, biased NTP pools are a well known factor for mutagenesis [15], implying that some of the extension products, which were used for assessing polymerase activity, are actually the products of mutation synthesis. Third, it is well established that cellular NTP concentrations range between 0.25–3 mM (µM, ATP, 3,152 +/− 1,698; GTP, 468 +/− 224; UTP, 567 +/− 460 and CTP, 278 +/− 242) [16].

In this report, we established new assay conditions that use unbiased and biologically relevant NTP substrate concentrations and avoid the kinetic bias found in the currently available gel-based assays. Importantly, this adjustment of the assay conditions not only revealed the robust polymerase activity of IAV Pol complex, but also enabled us to investigate the enzyme fidelity of IAV Pol. Also, with this new assay condition, we were able to conduct a series of comprehensive biochemical analysis to study the impact of various reactions parameters such as temperature, metals and viral NP on the IAV Pol fidelity. Overall, our results suggest that IAV Pol is least error-prone when compared to two reverse-transcriptases, HIV-1 RT and MuLV RT and also to bacterial phage T7 RNA polymerase. Importantly, the biochemical finding of the high fidelity nature of IAV Pol introduces a new concept of the mechanistic relationship between IAV Pol fidelity and the IAV replication strategy which uniquely provides IAV Pol with ample opportunities to generate genomic mutations during the multiple rounds of cRNA and vRNA amplification in a single infection cycle.

## Materials and Methods

### Expression and purification of influenza A virus RNA polymerase complex and other polymerases

For IAV polymerase, the PA, PB1 and PB2 genes (codon-optimized, Geneart) were amplified by reverse-transcription PCR from the H3N2 IAV strain (A/chicken/Nanchang/3-120/01), obtained from Dr. R. Webster, St. Jude Children's Research Hospital, Memphis, TN, USA. They were then cloned to the baculovirus expression vector pVL1392 (Invitrogen) between the *Bgl*II and *Xba*I sites. The PA gene was tagged at the C-terminus with the tandem affinity purification (TAP) tag, which consisted of a thrombin cleavage site followed by 6X His tag, tobacco etch virus (TEV) cleavage site and finally an IgG-binding domain. Each of the three pVL1392 plasmids were individually transfected into Sf9 insect cells for the production of recombinant viruses in SF900II SFM media (GIBCO), following the protocols provided by the vendor (BD Biosciences Pharmingen). To prepare the purified trimeric IAV polymerase, the three recombinant viruses were used for coinfecting Tni insect cells grown in Express Five SFM media supplemented with L-Glutamine (GIBCO). Cells were harvested 72 hours post infection and the lysates obtained were purified by the TAP purification technique as described previously [12], [17]. Typically, 1l expression culture produced 0.7 mg of the purified complex with approximately 95% purity, which was qualitatively compared with the band intensity of BSA with 95% purity (Sigma-Aldrich).The approximate molar ratio of PA:PB1:PB2, which was estimated by the protein band intensity in SDS-PAGE, was 1.8∶1∶1. T7 RNA polymerase was purchased from Ambion, and HIV-1 and MuLV RT proteins were isolated in our previous study [18].

### Overexpression and purification of H3N2 NP in *E. coli*

The H3N2 Nanchang NP gene (A/Chicken/Nanchang/3-120/01) was cloned to pET28a (Novagene), expressing NP protein fused to the six histidine tag at its N-terminal end. NP was purified using Ni^++^ column chromatography following the protocol provided by the vendor (Novagen). Fractions containing NP were pooled and dialyzed to a low salt buffer (50 mM Tris-Cl (pH 7.5), 1 mM EDTA, 200 mM NaCl, 10% glycerol, 1 mM DTT). The dialyzed protein was further purified using a second DEAE-Sephacel column to minimize any RNase contamination. The RNA binding of the purified NP was examined by a filter binding assay employing both protein and nucleic acid binding filters (data not shown).

### Template preparation

5′ vRNA (5′ AGUAGAAACAAGGCC 3′) and the 14-nucleotide (nt) 3′ vRNA which serves as a template (5′ GGCCUGCUUUUGCU 3′) from the PA gene of influenza A virus were purchased from Dharmacon. The other two templates (50-nt and 137-nt) used encode the first 50-nt and 137-nt sequences from the 3′ end of the Nanchang PA gene. The 50-nt template was purchased from Integrated DNA Technologies. The 137-nt RNA template was prepared by *in vitro* transcription using a double stranded (ds) PCR product amplified by a 37-mer forward primer encoding T7 promoter sequence and 19-nt of the viral sequence and a 16-mer reverse primer encoding 3′ sequence of the PA gene. The PCR product was used for synthesizing the 137-mer RNA template using *in vitro* transcription kit (Ambion), which was purified as described [19].

For the IAV Pol misincorporation reactions, a 30-nt RNA template (5′ GCAUUGUCGCAAUCAGUACCUGCUUUCGCU 3′) encoding the first 30-nt sequence from the 3′end of the PA gene was used (Integrated DNA Technologies). The T7 template sequence (5′ TAATACGACTCACTATAGGCGAAAGCAGGTACTGATTGCGACAATGC 3′) contains the T7 promoter region which is the first 18 nucleotides and the remaining 29 nucleotides (see the sequence underlined) are the same sequence as that of the flu template. The first A nucleotide is deleted because T7 pol requires G at the +1 site. This also explains why the T7 bands run one nucleotide lower than the IAV Pol reaction bands on the PAGE. For the HIV-1 RT and MuLV RT reactions, a 48-nt RNA template is used. This 48-mer RNA template (5′ GCAUUGUCGCAAUCAGUACCUGCUUUCGCUCUGGCUUAAGGGCGAUCG 3′) encodes not only the same first 30-nt sequence of the PA gene as the 30-mer Flu Pol template, but also the 18-nt non-PA gene which is annealed to a 18-mer DNA primer containing the A–G sequence at its 3′ end.

### ApG initiated RNA transcription

**Figure 1:**
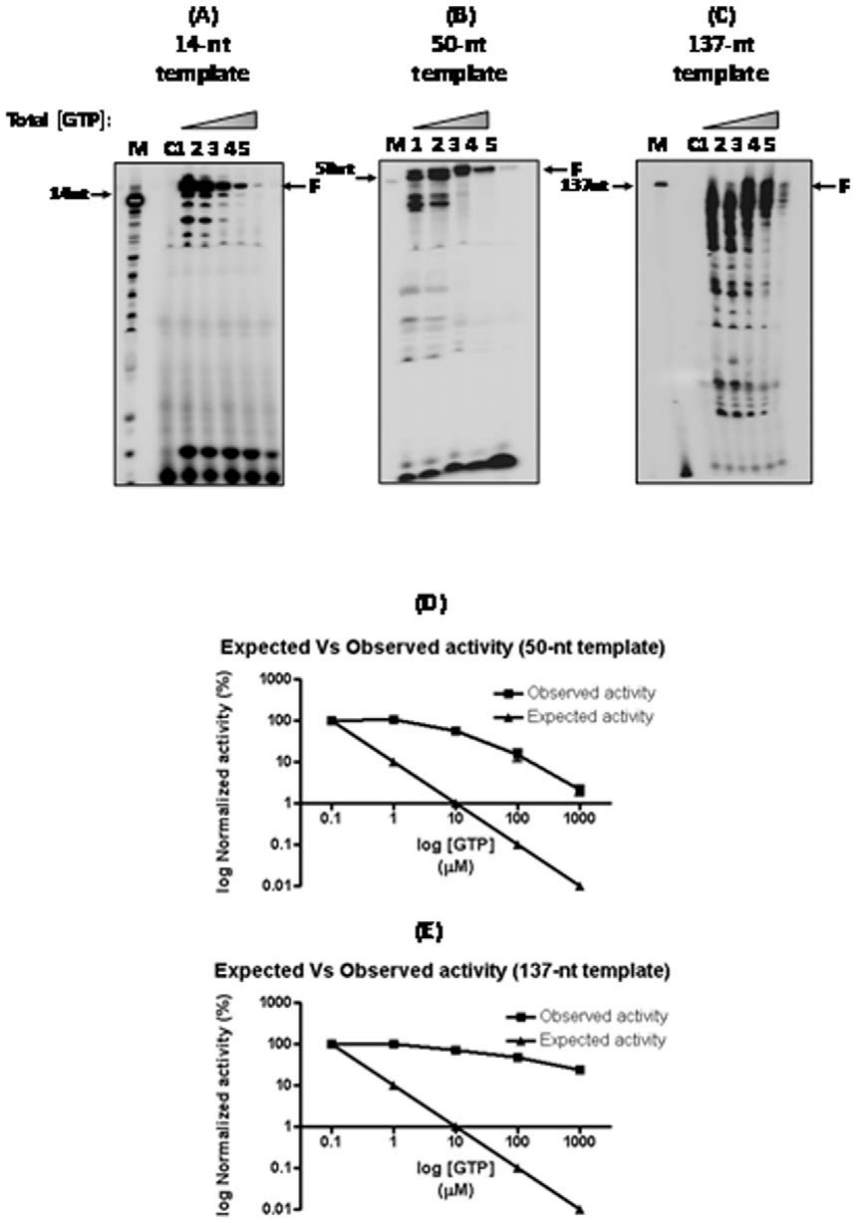
NTP concentration dependent ApG-primed RNA polymerase activity of IAV Pol complex. ApG primer (0.3 mM) was extended by IAV Pol using RNA templates encoding the first 14 (**A**), 50 (**B**) and 137 (**C**) nucleotides (nt) 3′ end sequences of the IAV PA gene. RNA polymerization was initiated by adding 1 mM ATP, 1 mM UTP, 1 mM CTP, 0.08 µM α-^32^P-GTP, and varying concentrations of nonradioactive GTP (lanes 1-5; 0.1, 1.0, 10, 100 and 1000 µM). **M**: Size markers **C**: Negative control without polymerase. **F**: Fully extended products. The fully extended products observed in the reaction with 50-nt (**D**) and 137-nt (**E**) were determined by densitometric analysis (observed activity) and these values were plotted against the actual GTP concentrations used in each reaction. The activity at 0.1 µM GTP was set arbitrarily to 100%.

We tested the transcriptional activity of the purified polymerase complex using the ApG transcription assay in which the 3′ and 5′ vRNA and the dinucleotide primer, ApG (Biosynthesis) are provided. ApG was extended using the 3′ vRNA template (14-nt) by the IAV polymerase (200 ng), generating a 14-nt long RNA fragment product. The ApG transcription reactions were performed as described elsewhere [20] at 37°C in 5 µl reactions mixtures containing transcription buffer (25 mM Tris-Cl, pH 7.5, 100 mM KCl, 5 mM Mg(OAC)_2_, 0.1 mM EDTA, 2 mM DTT, 0.25 µl Nonidet P-40, 12.5% glycerol), IAV Pol complex, 0.25 U/µl RNasin, 0.3 mM ApG, 1.6 µM 14nt 3′vRNA, 1.6 µM 5′vRNA, 0.08 µM radioactive α-^32^P-GTP (0.125 µl GTP, 3000 Ci/mmole, Perkin Elemer), and varying concentrations of nonradioactive NTPs (ATP, CTP, GTP and UTP) as described in figure legends. Transcription reactions were stopped after 1 h by the addition of 5 µl trichloroacetic acid (TCA) dye, denatured at 95°C for 3 min and transcription products were separated by 8 M urea denaturing 18–20% polyacrylamide gel electrophoresis (PAGE). The dried gels were analyzed by autoradiography using a PhosphorImager (Molecular Dynamics) and the intensity of the fully extended products was quantitated by OptiQuant (Version 3.10, Packard Instrument). In order to validate the linearity of the radioactive signals in this assay, we determined the signal intensity of the samples diluted by known ratios (i.e. 1, 1/2, 1/4, 1/8 and 1/16), and used that for all subsequent analysis of the signals. In the reactions described in Figure 1 which used varying amounts of radioactive and nonradioactive GTP, the ApG-primed reactions were performed in the presence of every 10-fold increasing concentrations of the nonradioactive GTP (0.1, 1, 10, 100 and 1000 µM) with 0.08 µM of radioactive α-^32^P-GTP and 1 mM of other three nonradioactive NTPs (ATP, CTP and UTP).

### Misincorporation assay

**Figure 2:**
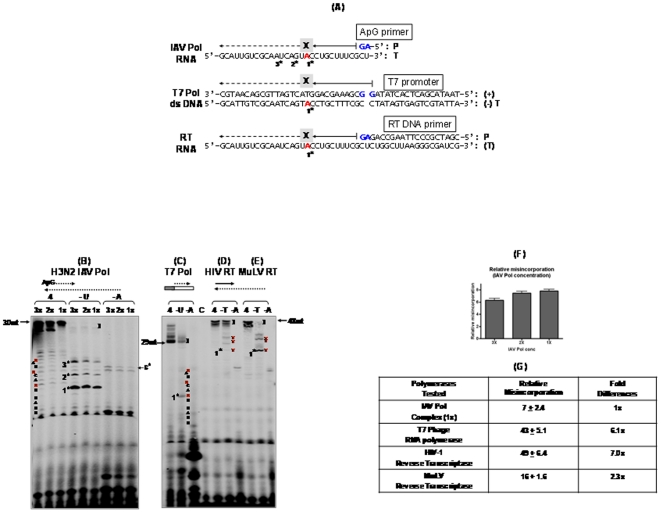
Misincorporation assay with IAV Pol, bacterial phage T7 RNA polymerase, HIV-1 RT and MuLV RT with biased nucleotide substrate pools. (**A**) Template sequences used for the misincorporation assay with IAV Pol, T7 RNA polymerase and RTs of HIV-1 and MuLV. The IAV Pol template used is a 30-nt sequence from the 3′ end of the viral PA sequence with a ApG primer binding site (P:primer, T:template,). The first UTP or TTP incorporation site of each template was marked with “X”, and the first stop site in the (−) UTP or TTP reaction was marked with “1*” under each template sequence (the second and third stop sites were marked “2*” and “3*” for the IAV template). The RNA synthesis initiation sites were marked in blue. (**B**) ApG-initiated RNA-dependent RNA polymerization by IAV Pol: ApG primer was extended with a 30-nt RNA template and three different amounts of H3N2 IAV Pol protein (1x, 2x and 3x) with 500 µM four NTPs (“4”) and 0.16 µM α-^32^P-GTP, and the same reactions were repeated except using two biased NTP pools, minus UTP (“- U”) or minus ATP (“- A”). The sequence of the incorporated nucleotides near the three UTP stop sites (red) is shown at the side. The extended products in the (−) UTP reaction, which was used for calculating the misincorporation efficiency, was marked as “]”.Dotted lines refer to RNAs. (**C**) DNA dependent RNA polymerization of bacterial phage T7 RNA polymerase: A 47 bp ds DNA (box) encoding T7 promoter (grey box) and 29 bp sequence (white box) was used for RNA synthesis with T7 RNA polymerase at 37°C for 60 mins. “]”: Fully extended misincorporated product. (**D**) and (**E**) RNA dependent DNA polymerization reaction by HIV-1 RT (D) and MuLV RT (E). A 48 mer RNA template annealed to a 18-mer single stranded DNA primer was used for the DNA polymerization by HIV-1 and MuLV RTs at 37°C for 60 mins. 500 µM dNTPs mixed with α-^32^P-dNTPs (0.16 µM), which is the same nucleotide concentration and ratio as used in the reactions with IAV Pol and T7 RNA polymerase, was used for DNA synthesis. “]”: Fully extended misincorporated product. Dotted lines refer to RNA and solid lines refer to DNA. (**F**) and (**G**) Comparison of the misincorporation efficiency of the four polymerases. For calculation of the misincorporation efficiency, the fully extended misincorporated product in (−) U/(−) T reactions in all four polymerases was normalized to the total extended product in the lanes with all four NTPs (dNTPs). The fold differences of the calculated misincorporation percentages between three activities of IAV Pol (**F**) and between IAV Pol and three other polymerases (**G**) were determined. At least five repeats of the assay were conducted in this analysis.

In the misincorporation assays with all four polymerases, an equal concentration of all the nucleotides (NTPs or dNTPs) was provided using a mix of four α-^32^P-NTPs or dNTPs (0.16 µM, PerkinElmer, specific activity, 3000 Ci/mmol) and nonradioactive NTPs or dNTPs (500 µM). For IAV Pol, the ApG-transcription reactions were performed with the 30-nt 3′ RNA template as described (Figure 2A), and the reactions were repeated with only three kinds of NTPs after deleting ATP [(−) A] or UTP [(−) U]. Similarly, for the T7 RNA polymerase reactions, 2 µl of normalized protein (Ambion) was mixed with 50 nM ds DNA template containing T7 promoter (Figure 2A) in the provided reaction buffer at 37°C for 1 hr. For HIV and MuLV RT, a mixture of 200 nM 48-mer RNA template annealed to 100 nM 18-mer DNA primer (Figure 2A), 500 µM dNTPs, 0.16 µM α-^32^P-dNTPs (PerkinElmer, 3000 Ci/mmol) were incubated with RT for 1 hour at 37°C under conditions described previously (16). In the (−) A and (−) T reactions, dATP and dTTP were left out, respectively.

**Figure 3:**
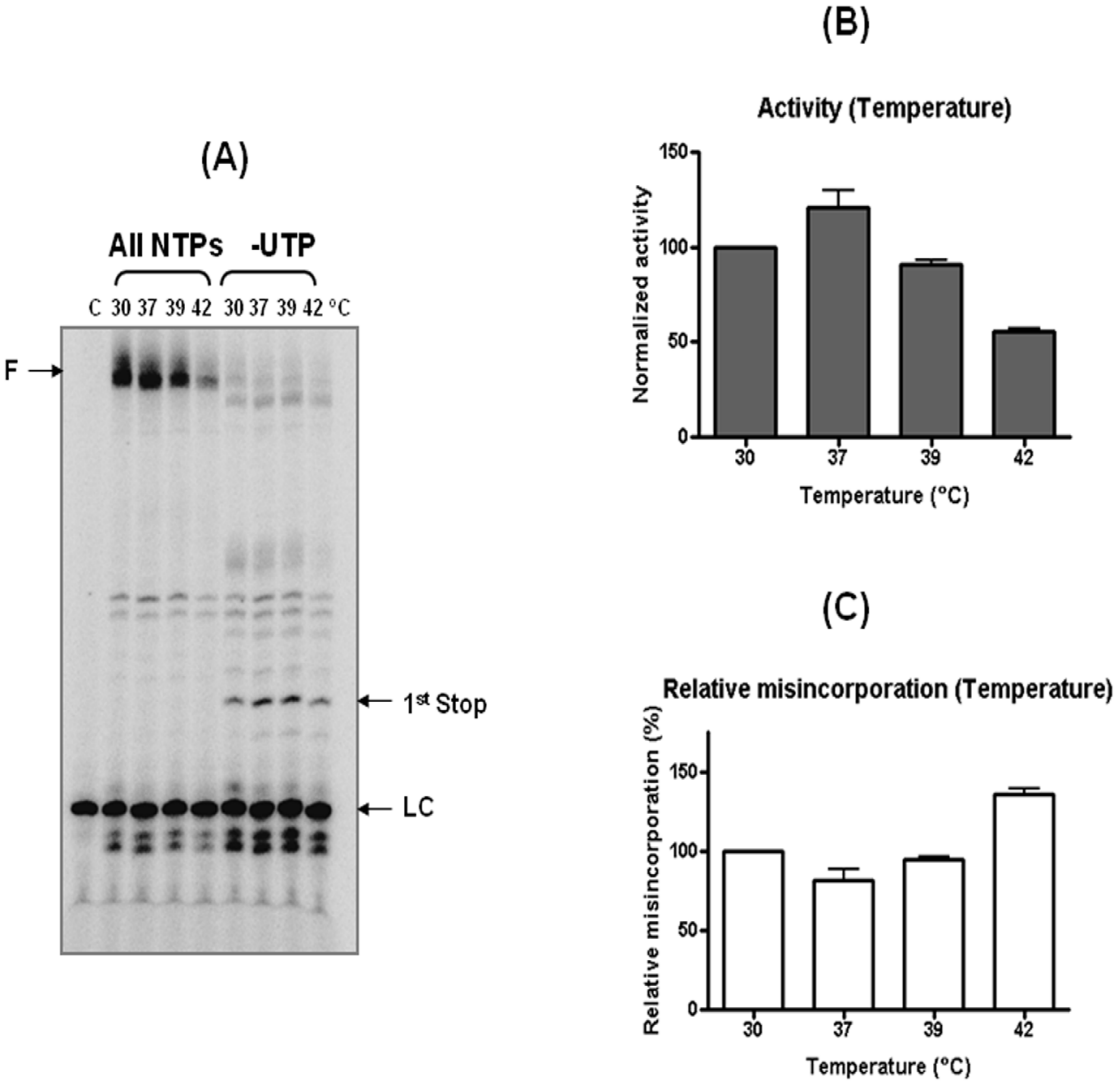
Effect of temperature on polymerase activity and misincorporation efficiency of IAV Pol complex. (**A**) IAV polymerization at different temperatures. LC: loading control (**B**) Fully extended products for the polymerase activity of IAV Pol at the four different temperatures were compared after being normalized with the value obtained at 30°C (100%). (**C**) The misincorporation efficiency of IAV Pol at four temperatures were calculated as described in Figure 2F, and compared with the value obtained at 30°C (100%). C: no polymerase control.

For further biochemical characterization, misincorporation reactions were carried out at various temperatures (30, 37, 39 and 42°C). For testing the effect of Mn^++^, Mg(OAC)_2_ (5 mM) in the buffer was replaced with varying concentrations of MnCl_2_ (0.5, 1, 5, 7.5 mM). Further, the reactions were performed with and without different amounts of purified NP. For reactions with NP, NP was preincubated with the RNA template prior to the addition of IAV Pol in the reaction mixture. Note that to minimize any loading variations between reactions, which can affect the total activity measurement, we added an equal amount of a 5′ end ^32^P-labeled non specific primer to each reaction, and used this as the loading control (i.e. “LC” Figure 3). The transcription products in all the misincorporation reactions were separated by 8 M urea denaturing 18–20% PAGE.

## Results and Discussion

### RNA polymerase activity of IAV Pol complex using biologically relevant NTP concentrations

First, in order to address the potential kinetic bias induced by the biased NTP pools that have been typically used in the gel-based IAV Pol assays, we conducted the ApG-initiated IAV RNA polymerase reaction using gradually increasing concentrations of the limited NTP (GTP) in the reaction, which abolishes the NTP pool bias (Figure 1). The ApG-primed reactions were performed in the presence of every 10-fold increasing concentrations of the nonradioactive GTP (0.1, 1, 10, 100 and 1000 µM) with a 0.08 µM of radioactive α-^32^P-GTP and 1 mM of other three nonradioactive NTPs (ATP, CTP and UTP). The polymerase was expressed and purified from the baculovirus system as we described previously [17]. The reaction mixtures contained the Nanchang H3N2 IAV Pol complex, ApG (0.3 mM), 5′ RNA 15-mer (1.6 µM), and one of three different 3′ RNA templates, 14-nt (Fig. 1A), 50-nt (Fig. 1B) and 137-nt (Fig. 1C) encoding the 3′ end sequence of the Nanchang IAV PA gene. The reactions were performed at 37°C for 60 min, and were analyzed by denaturing 8 M urea-PAGE. The extent of the fully extended products, which is indicative of the polymerase activity in the multiple nucleotide incorporation assays, was determined.

Importantly, note that the actual portion of the radioactive α-^32^P-GTP proportionally decreases upon mixing with every 10-fold increase in concentration of the nonradioactive GTP (0.1, 1, 10, 100 and 1000 µM, lanes 1 to 5 in Fig. 1). Thus, the NTP bias was abolished in the reaction with 1000 µM nonradioactive GTP where the portion of the radioactive GTP (0.08 µM) is minimal in the final GTP concentration (1000.15 µM). If the polymerase activity remains unchanged under this condition, the radioactive GTP incorporation in the primer extension, which can be visualized and quantitated from the radioactive band intensity of the extended products, should proportionally decrease, due to the increasing probability of the nonradioactive GTP incorporation instead of α-^32^P-GTP. However, as shown in Figure 1, in all of the reactions with the three different RNA templates, we did not observe the predicted proportional 10-fold reduction of the fully extended products (14-nt, 50-nt and 137-nt), implying that the polymerase activity of the Pol complex was actually elevated when the total GTP concentration increased. This observation with the different sized templates indicates that the effect of GTP concentration on the activity of the enzyme is consistent, regardless of the sequence and size of the template.

Next, we plotted the actual extent of the full length products in the 50-nt (Fig. 1D) and 137-nt (Fig. 1E) template reactions, which corresponds to the total IAV Pol activity in each reaction. The observed activity is then compared with the expected activity i.e., the predicted product extent which should decrease ten times with every 10-fold increase of nonradioactive GTP concentration. As seen in the plots of the data from the 50-nt (Fig. 1D) and 137-nt products (Fig. 1E), the total IAV Pol activity measured from the gel analysis was significantly higher than the expected activity. Strikingly, fully extended products were able to be detected in the reaction with an equal 1000 µM of all four NTPs (Fig. 1, lanes 5) where only ∼0.01% of the total GTP was radioactive. Thus, this data suggests that the polymerase activity of H3N2 IAV Pol must have been elevated at least 200–500 times when we used a 1000 times higher concentration of the final GTP than the concentration that has typically been used in various recent studies. This observation also implies that the IAV Pol activity has been underestimated in the IAV Pol assay that employed limited GTP because the reaction rate at this limited GTP pool is likely below Vmax.

### Misincorporation efficiency of IAV Pol complex

It has been well-established that a biased nucleotide pool is a major factor to promote polymerase-induced mutation synthesis [21]. Basically, polymerases pause at the template sequences where the limited nucleotides are supposed to be incorporated, and the temporal pausing of the polymerase triggers the incorporation of an incorrect nucleotide, called misincorporation, which eventually generates mutations. Therefore, another important indication of the data presented in Figures 1 would be that the full extended products observed in the significantly biased NTP pools could include the products of misincorporation to some extent, particularly at low total GTP concentrations (Fig. 1, lanes 1).

In fact, one commonly used assay to investigate the enzyme fidelity of polymerases is a primer-extension based misincorporation assay employing completely biased nucleotide pools that consist of only three of nucleotides instead of all four nucleotides [22], [23], [24]. Under this complete nucleotide bias condition, the primer extension pauses at one nucleotide before the template sequence that the deleted nucleotide is supposed to be incorporated, called a “stop site”. However, low fidelity enzymes tend to efficiently incorporate incorrect nucleotides at the pause sites, and display the efficient primer extension products beyond the stop sites, whereas high fidelity enzymes exhibit little primer extension beyond the stop sites. The efficiency of the primer extension beyond the first stop site in the total primer extension has been used to compare the enzyme fidelity between polymerases [5], [22], [25].

For this test, we performed the 30-nt template-based and ApG-initiated polymerization reactions in which a mixture of all four NTPs (or three in case of misincorporation reactions) are provided. The ratio of radioactive NTP to nonradioactive NTP is kept constant for all the NTPs and is approximately 1/1600, allowing us to use a low concentration of radioactive NTPs. The 30-nt template also encodes the 3′ end 30-nt sequence of the viral PA gene template (Figure 2A). As shown in Figure 2B, in the reaction with all four NTPs (500 µM), H3N2 IAV Pol complex extended the ApG primer and generated the 30-nt long fully extended products, and the extents of the fully extended products were proportional to the amounts of the IAV Pol complex under the reaction conditions (1x, 2x and 3x: Fig. 2B, 3X = 200 ng), confirming that the NTP incorporation kinetics was linear under the employed polymerase reaction. Upon the deletion of UTP or ATP from the four NTPs [(−) U or (−) A in Fig. 2B], however, the amount of fully extended products were significantly reduced, indicating that the biased NTP pool generated major kinetic blockages in the IAV Pol polymerization reaction. As predicted, the first “stop site” was observed at one nucleotide before the sites where the deleted UTP (“X” in Fig. 2A) should be incorporated (Fig. 2A and 2B: see “1*” in the (−) U reactions of and the sequences of the expected products). Excitingly, primer extension was observed beyond the first stop site, generating the second (“2*”) and third (“3*”) stops sites (Figure 2A and 2B). This indicates that mutation synthesis must have occurred at not only the first stop site but also the later stop sites.

Unlike the UTP stop sites, it is difficult to observe the first stop sites induced by the deletion of other three NTPs (A, G and C) because the first 5 nucleotides in the 3′ end conserved sequence of the IAV genomes contain A, G and C. Thus, their first stop sites generate only 3–5 nucleotide long products which are technically difficult to separate even with 8 M urea high percentage denaturing PAGE. However, as shown in the reactions with (−) ATP pool (Fig. 2B), the full length extension was also significantly decreased which supports that the biased NTP pool generates kinetic blockage. Interestingly, the sixth “A” incorporation site is present at the 18th nucleotide from the 3′ end of the 30-nt template sequence and this could be observed in the (−) ATP reaction (“6*”) in Figure 2B. The product seen at the sixth A incorporation site must have been generated after IAV Pol made mutations at the five different stop sites prior to the fifth sixth stop site seen in the gel. Importantly, we observed that the reactions with (−) CTP or (−) GTP biased pools also induced the reduction of the fully extended product (data not shown). Indeed, this is the first biochemical observation reported for mutation synthesis by IAV Pol.

### Misincorporation efficiency of bacterial T7 RNA polymerase, HIV-1 RT and MuLV RT with templates encoding identical sequences

Next, we compared the enzyme fidelity of IAV Pol complex with three other polymerases, bacterial phage T7 DNA-dependent RNA polymerase as well as two retroviral RNA-dependent DNA polymerases which are well-characterized for their fidelity [26]: 1) murine leukemia virus reverse transcriptase (MuLV RT) with high fidelity and 2) human immunodeficiency virus Type 1 reverse transcriptase (HIV-1 RT) with low fidelity [18], [24], [26], [27] Importantly, these polymerases initiate the nucleic acid synthesis by using their own unique template and primer complexes (i.e. double stranded DNA with promoter sequence for T7 RNA polymerase, and a DNA primer annealed to a RNA template for RTs) as illustrated in Figure 2A. First, we conducted a RNA synthesis reaction with T7 RNA polymerase under the same NTP concentration and ratio between radioactive and nonradioactive GTP (1/1600) as the IAV reaction except for the template sequence. As illustrated in Figure 2A, the T7 Pol template used is a 47-bp double stranded DNA (Fig. 2C) containing 18-bp T7 promoter and 29-bp of the sequence which is the same as the IAV RNA Pol template. The first nucleotide of the RNA product starts from G instead of A because T7 Pol prefers G as the initial nucleotide in RNA synthesis [28]. We also used the T7 Pol activity which displayed a similar level of the fully extended product as the IAV Pol reaction (Fig. 2B). As shown in Figure 2C, the first (−) UTP stop site was observed in the T7 Pol reaction (“1*” in Fig. 2C). However, a significantly high level of the fully extended product was observed (see “]” in Fig. 2C), compared to the IAV Pol reaction with (−) UTP (see “]” in Fig. 2B).

Next, we conducted the (−) dTTP and (−) dATP misincorporation assay with low fidelity HIV-1 and high fidelity MuLV RT proteins. The RT reactions employed a 48-nt RNA template encoding the 3′ end 30-nt sequence of the PA gene and 18-nt non-viral sequence annealed to an 18-nt DNA primer (Fig. 2A). Thus, the single stranded 30-nt PA sequence of the 48-mer RNA template where DNA synthesis is catalyzed by RTs encodes the exactly same 30-nt sequence as the influenza Pol 30-nt template used in Figure 2B. The RT reactions were conducted using the same nucleotide substrate (dNTP) concentration and the same ratio between nonradioactive and radioactive dNTP (1/1600) as the IAV Pol and T7 RNA Pol reactions. First, we normalized the polymerase activity of HIV-1 and MuLV RTs showing a similar band intensity of the fully extended product with all four NTPs as the full length bands in the 1x activity of the IAV Pol reactions (Fig. 2B). Importantly, note that the specific activities of the α-^32^P-NTP and α-^32^P-dNTP, which were used in the IAV Pol and RT reactions, respectively, were identical (3000 Ci/mmole), and thus the same band intensity of the fully extended products in the reactions with different polymerases indicates the same level of nucleotide incorporation and nucleic acid synthesis.

Next, we repeated the same RT reactions with the equal activity of low fidelity HIV-1 and high fidelity MuLV RTs but with the (−) TTP or (−) dATP biased pools. Indeed, HIV-1 RT displayed much more extension beyond the first stop site (“1*”: see the sites marked with “U” in Figure 2D) and full length products (see “]” in Fig. 2D) than MuLV RT (Fig. 2E). This observation not only confirms that HIV-1 RT is more error-prone than MuLV RT, but also validates the fidelity assay condition established in this study. We also confirmed the first stop sites in these RT reactions using the same primer extension reactions but with 5′ end γ-^32^P-labeled DNA primer and four different biased dNTP pools (data not shown).

Now with the misincorporation assays completed with the three other polymerases, we were able to compare their enzyme fidelity by comparing the extent of the primer extension beyond the first stop site (see “*” in Fig. 2). First, as seen in Figure 2B (1x) for IAV Pol with (−) UTP and Figure 2D for HIV-1 RT with (−) TTP, IAV Pol generated much shorter and fewer RNA products beyond the first stop site than HIV-1 RT even if there was a 2-fold more total polymerase activity of the 1x IAV Pol than HIV-1 RT, which was estimated from the reaction with all four nucleotides present. This suggests that IAV Pol complex has a lower misincorporation capability than HIV-1 RT. Also, HIV-1 RT showed much higher extension beyond the first stop site (Fig. 2D) than MuLV RT (Fig. 2E), suggesting that HIV-1 RT has lower fidelity than MuLV RT, which has been demonstrated previously (25). Interestingly, IAV Pol complex generated similar or slightly less primer extension beyond the first stop site than the high fidelity MuLV RT even if a lower polymerase activity of MuLV RT, compared to IAV Pol, was used, supporting that IAV Pol complex has at least similar fidelity with MuLV RT. Overall, the data shown in Figure 2 qualitatively suggests that IAV Pol complex is a high fidelity enzyme.

It is important to address that IAV Pol may fall off from the template during the initial synthesis [14], which can induce the pausing of the primer extension reaction, and this is also true for several RNA polymerases using the promoter such as T7 RNA polymerase used in this study [29]. Since our study observes the pausing events at 11–13 nt away from the first nucleotide incorporation, the intrinsic pausing nature of these polymerases at the initiation sites may be minimal. However, it is still possible that some fraction of the pauses observed in our study may result from the pausing induced during the initiation of RNA synthesis. In addition, there are a number of other factors known to affect the processivity of polymerases such as template sequences, template RNA secondary structures, substrate availability, salt, and temperature. In this study, we employed identical template sequences for all four enzymes to minimize any template-induced pausing.

### Quantitative comparison of the misincorporation efficiency of IAV Pol complex with T7 RNA polymerase, HIV-1 RT and MuLV RT

Finally, we estimated the fidelity difference between IAV Pol and three other enzymes analyzed in Figure 2. Since in the assay described in Figure 2, the misincorporation level is proportional to the total polymerase activity used, we could simply calculate the misincorporation efficiency in the (−) UTP (or TTP) reactions with each of the four polymerase reactions by normalizing the band intensity of the total misincorporated product (“]”) of the (−) UTP (or TTP) reaction with the band intensity of the total polymerase activities used which is represented by the full length products in the all four nucleotide reactions. First, we examined if the misincorporation efficiency remains similar in the reactions with three different activities of IAV Pol (1x, 2x and 3x: Fig. 2B). As shown in Figure 2F, the misincorporation efficiency of IAV Pol was relatively constant regardless of the IAV Pol activity used, and this validates that the polymerase activity and reaction conditions employed in Figure 2 are proper for fidelity comparisons. Second, HIV-1 RT showed 3 times higher misincorporation efficiency than MuLV RT in this gel based assay. In fact, HIV-1 RT previously showed 4 to 10 times lower fidelity than MuLV RT in various fidelity assays (1, 29, 31) and this further validates our gel based assay system. With this validation, next, we calculated and compared the misincorporation efficiency of IAV Pol with the three other polymerases. As summarized in Figure 2G, indeed, IAV Pol is 7 and 2.3 times less error-prone than HIV-1 and MuLV RTs, respectively, and 6.1 times less error-prone than T7 RNA polymerase. Importantly, since all of the four polymerases used in Figure 2 lack 3′ to 5′ proof-reading activity, the mutation synthesis observed in the misincorporation assay must be catalyzed only by their polymerase active sites.

### Effect of temperature, Mn^++^, and influenza nucleoprotein (NP) on the misincorporation efficiency of IAV Pol complex

Previous studies with various DNA and RNA polymerases demonstrated that the enzyme fidelity of DNA polymerases is highly sensitive to reaction conditions such as metals [30], [31] and reaction temperature [32], [33]. Next, by using the physiological NTP condition showing robust activity of IAV Pol and the misincorporation assay established in Figure 2, we investigated a series of comprehensive biochemical characterizations for the effects of the temperature, Mn++ and viral RNA binding protein, NP, on the polymerase activity and fidelity of IAV Pol. First, we examined the effect of the temperature on the IAV Pol fidelity. The ApG-initiated RNA synthesis was conducted in the presence of all four NTPs or (−) UTP mix by the IAV Pol complex at 30, 37, 39 and 42°C for 1 hr. Note that to minimize any loading variations between reactions, which can affect the total activity measurement, we added an equal amount of a 5′ end ^32^P-labeled non specific primer to each reaction, and used this as the loading control (“LC” Figure 3). The fully extended products in the reactions with all four NTPs were used for estimating the temperature dependence of IAV RNA polymerase after being normalized with the loading control (LC) band intensity. As shown in Figure 3A and 3B, the H3N2 IAV Pol complex showed the highest polymerase activity at 37°C and its activity largely decreased at 42°C. However, while the polymerase activity significantly reduced at 42°C, the level of the misincorporation beyond the 1^st^ stop site at 42°C remained similar with the reactions at lower temperatures. Since the misincorporation capability was defined as the ratio of the level of the primer extended beyond the first stop site with the biased NTP pool to the total polymerase activity measured in the reaction with all four NTPs, the misincorporation capability of IAV Pol actually increased at 42°C, compared to that at the lower temperatures. Indeed, when the misincorporation efficiency at each temperature was calculated (Fig. 3C), the IAV Pol complex was more error prone at 42°C compared to the low temperatures. In fact, this temperature effect of the IAV Pol fidelity is novel because our previous study with RT proteins showed elevated fidelity at high temperatures such as 55°C [32]. In addition, the optimal temperature for the IAV Pol activity varies among viral strains as we recently reported [12], and avian strain Pol complexes appear to display its optimal activity at higher temperature in general [34].

**Figure 4:**
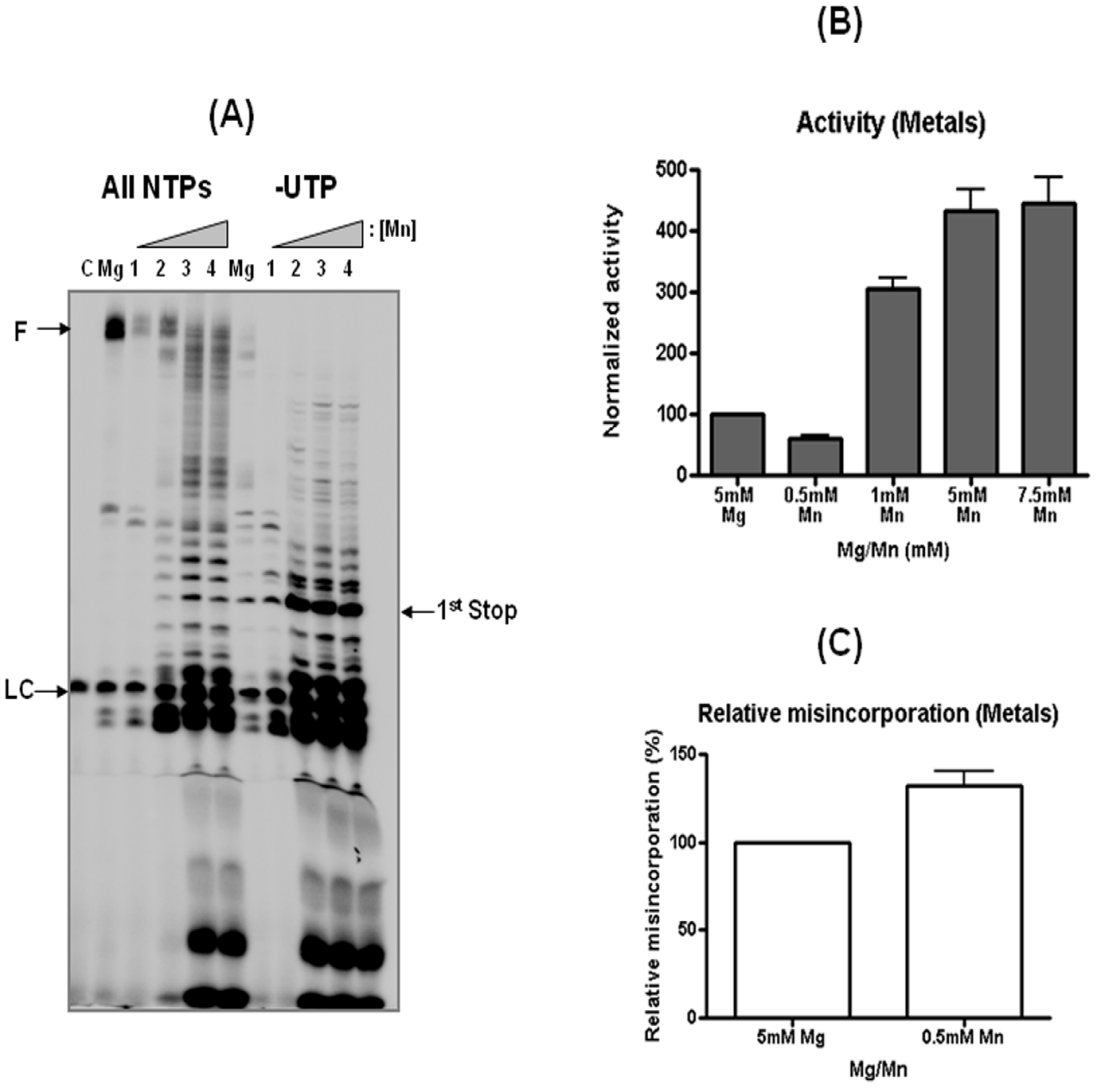
Effect of manganese on polymerase activity and misincorporation efficiency of IAV Pol complex. (**A**) IAV polymerization with 5 mM Mg(OAC)_2_ and varying concentration of MnCl_2_ (lanes 1 to 4, 0.5, 1, 5, 7.5 mM). LC: loading control (**B**) Fully extended products for the IAV Pol polymerase activity with different metal concentrations were compared after being normalized with the value obtained with 5 mM Mg(OAC)_2_ (100%). (**C**) The misincorporation efficiency of IAV Pol at 0.5 mM MnCl_2_ was calculated as described in Figure 2F, and compared with the value obtained with 5 mM Mg(OAC)_2_ (100%). C: no polymerase control.

Mg^++^ is a common metal ion used by DNA and RNA polymerases which essentially facilitates the nucleophilic attack of the 3′ OH at the primer to the α-phosphate of the incoming nucleoside triphosphate to form a phosphodiester bond. It has been shown that replacing Mg^++^ with Mn^++^ promotes mutation synthesis in some DNA polymerases [35]. First, we examined the effect of Mn^++^ on the polymerase activity of H3N2 IAV Pol. 5 mM Mg^++^, which is commonly used in the IAV Pol assay, was used a control (Figure 4A), and the same reaction was repeated with varying Mn^++^ concentrations. As shown in Figure 4A and 4B, the H3N2 IAV Pol showed much lower polymerase activity with 0.5mM Mn^++^ than the reaction with 5 mM Mg^++^. Interestingly, at 1, 5 and 7.5 mM Mn^++^ the IAV Pol actually showed increased total NTP incorporation than with 5 mM Mg^++^. However, the primer extension at 1, 5 and 7.5 mM Mn^++^ became highly distributive as shown by the accumulation of abortive products, suggesting that the processivity of IAV Pol significantly reduced at high Mn^++^ concentrations. A recent report which employed biased NTP pool conditions, also showed increased activity by increasing Mn^++^ concentration although the activity with Mn^++^ is much lower than Mg^++^ [36] Next, we calculated the misincorporation efficiency using the equation described above. For this, we used the concentration of Mn^++^ which showed comparable activity to 5 mM Mg^++^ i.e., 0.5 mM Mn^++^ without any processivity defect. Even after normalizing these two reactions for activity differences, the reaction with Mn^++^ shows higher misincorporation. Thus, this data supports numerous earlier reports in literature stating that Mn^++^ promotes mutation synthesis by inserting a higher ratio of incorrect to correct substrate. Also, it leads to decreased hydrolysis of the mismatched product which explains the abortive products in the figure [37].

**Figure 5:**
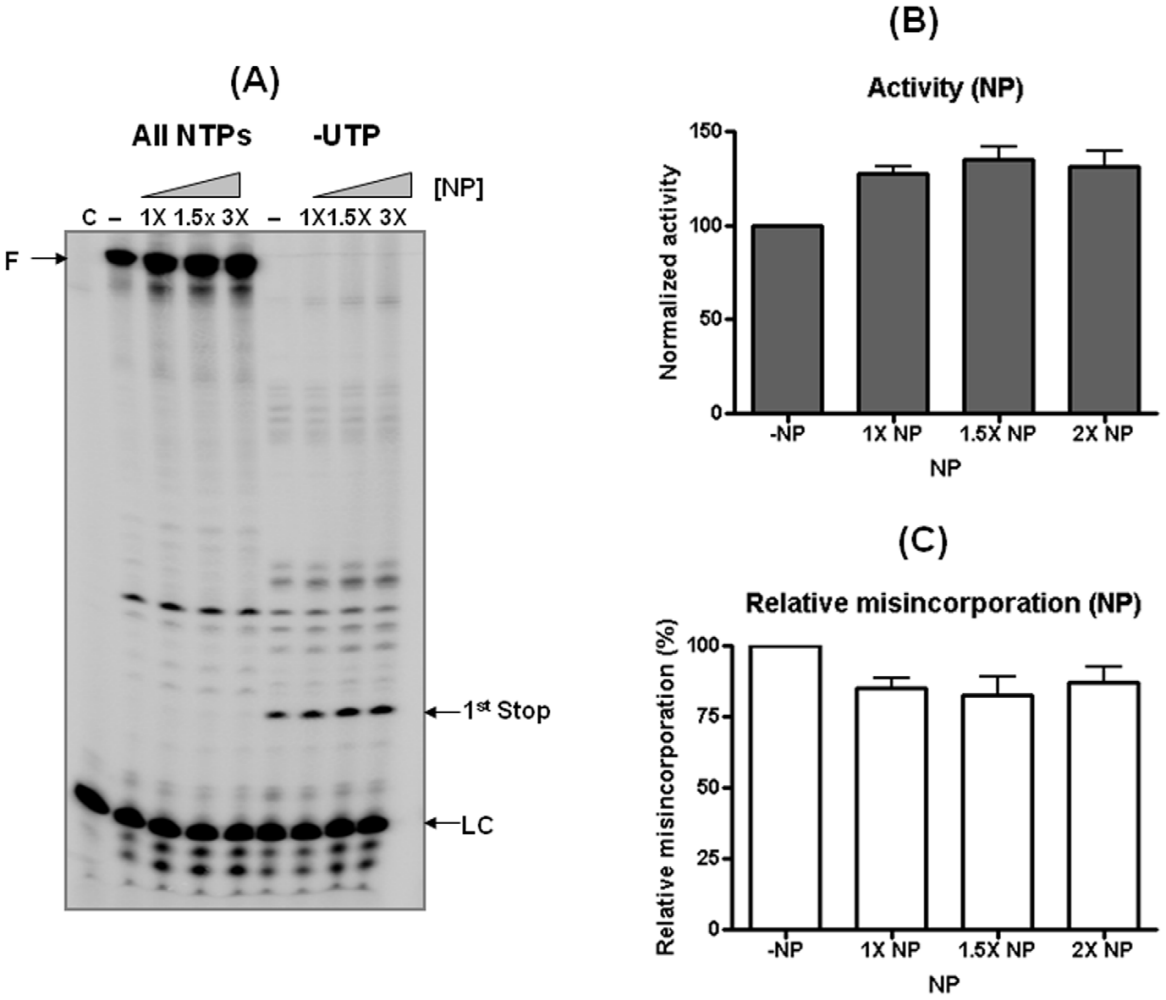
Effect of H3N2 NP on polymerase activity and the misincorporation efficiency of the IAV Pol complex. (**A**) IAV polymerization in the presence and absence of varying concentrations of H3N2 NP with the 50-mer template used in Figure 1B. Molar ratios between the RNA template and NP molecule were altered between 0 and 2. The RNA template was preincubated with NP before adding the polymerase complex. LC: loading control; (**B**) Fully extended products for the IAV Pol activity with different molar ratios of RNA/NP were compared after being normalized with the value obtained with no NP (100%). (**C**) The misincorporation efficiency of IAV Pol was calculated as described in Figure 2F, and compared using the value obtained with no NP (100%). C: no polymerase control.

Lastly, we investigated the effect of influenza virus NP which is known to both bind viral RNA template as well as interact with the IAV polymerase [38]. For this, we expressed and purified the H3N2 NP protein in *E. coli*. Since one single NP molecule binds to RNA every 24 nucleotides [38], the 30-mer RNA template used in the previous figures is too short for this test. Thus we used the 50-nt long RNA template which was used in Figure 1B. The IAV Pol reactions were conducted in the absence and presence of varying amounts of NP which can coat 100, 150 and 200% of the 50-nt RNA template. As shown in Figure 5A and 5B, the polymerase activity of the IAV Pol increases with increasing NP concentration, which has been previously observed [39]. However, upon the normalization with the total activity, the misincorporation efficiency decreased slightly in reactions with NP.

### Virological implications of the high fidelity nature of IAV Pol with respect to other RNA viruses

It has been well established that the enzyme fidelity of viral polymerases is a main determinant for viral genetic hyper-mutability and evolution; and in the case of HIV-1, the highly error-prone HIV-1 RT is considered as a key contributor for viral genomic hypervariability [5], [40]. This concept has been supported because alterations in the HIV-1 RT fidelity directly affect HIV-1 mutation rate [40]. Considering the high viral capacity of influenza viruses to evolve both within and between host species as well, the observation of the relatively high enzyme fidelity of the IAV Pol complex was rather unexpected. Interestingly, however, several primer extension based biochemical studies also reported that the RNA-dependent RNA polymerase of poliovirus, which is a positive strand RNA virus, is a high fidelity enzyme [41].

Thus, we reasoned about the disagreement between high genetic variability of IAV and relatively high enzyme fidelity of IAV Pol complex found in this report. IAV has a replication (RNA synthesis) strategy that amplifies their genomes many times per infection by using exclusively their own polymerase complex without any assistance from host RNA polymerases. This is contrasted to retroviruses (i.e. HIV-1) that replicate their single stranded RNA genomes into double-stranded proviral DNA in two ways. First, HIV-1 replicate their RNA genome to proviral double stranded DNA only twice per infection (conversion of (+) single strand RNA genomes to (−) single strand DNA and then conversion of the synthesized (−) DNA to double stranded proviral DNAs) by using their own polymerases. Second, the RNA genomes of every HIV-1 progenies are synthesized by host RNA polymerase II by transcribing the proviral DNA integrated into the host chromosomes. It is a reasonable assumption that the viruses, which require a high level of genomic mutations for their evolution and escape but have only a few chances to replicate their genomes by their own polymerases (i.e. HIV-1), might have evolved to harbor highly error-prone polymerases (i.e. HIV-1 RT) to generate sufficient mutations in really limited chances. In contrast, due to numerous rounds of the amplification of single viral RNA genomes to viral RNAs only by the viral polymerase, IAV Pol has ample chances to make and accumulate genomic mutations in each viral infection cycle. It has been estimated that the viral burst size in case of influenza, in one cycle of replication, is about 1000 [42], and thus influenza virus should undergo ∼1000 rounds of genome replication solely by viral RNA polymerase. If IAV Pol complex is highly error-prone, like HIV-1 RT, then this may cause the excessive production of unwanted genomic mutations, ultimately leading to catastrophic mutagenesis. Thus, it can be hypothesized that the high enzyme fidelity nature of IAV Pol complex which was observed in this study may enable the viruses to avoid this lethal hypermutagenesis. However, even with high enzyme fidelity, the multiple amplification replication strategy may allow the IAV to produce genomic mutations sufficient for viral evolution and host adaptation.

The similar reasoning of the high fidelity viral RNA polymerases envisioned above for IAV Pol can be applied to polioviruses which also amplify their RNA genomes multiple times per infection solely by their own high fidelity RNA polymerase. In addition, it was demonstrated that the elevation of genomic mutation rates of poliovirus, which is promoted by an antiviral agent (ribavirin), results in the catastrophic mutagenesis [41], [43], supporting the notion that poliovirus is vulnerable to hyper-mutagenesis, and thus their polymerase fidelity might have evolved to be a faithful RNA polymerase. Therefore, since IAV has similar genome replication strategy with poliovirus, a similar mechanistic relationship between the high fidelity nature of the IAV Pol complex and viral high genetic and evolutionary potentials can be applied to influenza A virus.

It is also important to address other potential cellular events that may generate IAV genomic mutations during viral life cycle. One potential mechanism well established is host enzymes that can chemically alter the structures of the nucleotide substrates. APOBEC3G is a host cytidine deaminase which can convert to C to U in the proviral DNA of HIV-1, and this event induce the G to A mutation in viral genomic DNA post reverse transcription, leading to lethal mutagenesis [44]. Interestingly, however, HIV-1 evolved to encode a unique accessory protein, Vif, which counteracts the APOBEC3G and escape from the lethal mutagenesis effect of APOBEC3G. Interestingly, while influenza virus infection elevates the APOBEC3G expression, no anti-viral activity was detected [45]. It was also reported that another type of host deaminase, APOBEC3F, appears not to display anti-influenza virus effect. In addition, various oxidated NTPs can be also incorporated during viral RNA synthesis, which may also affect IAV mutagenesis. Thus further studies on the mechanistic interplays of IAV evolution with the IAV Pol fidelity and other viral and host factors that can produce the IAV genomic mutations are necessary to firmly address the sources of the IAV genomic mutagenesis.
